# Comparison of Different Fixation Methods for Combined Histological and Biomolecular Analysis of Fixed and Decalcified Bone Samples

**DOI:** 10.3390/mps5040064

**Published:** 2022-07-21

**Authors:** Sarah Al-Maawi, Priscilia Valenzuela, Eva Dohle, Anja Heselich, Robert Sader, Shahram Ghanaati

**Affiliations:** Frankfurt Oral Regenerative Medicine (FORM-Lab), Clinic for Maxillofacial and Plastic Surgery, Goethe University, 60590 Frankfurt am Main, Germany; sarah.al-maawi@kgu.de (S.A.-M.); priscilia@valenzuela.de (P.V.); eva.dohle@kgu.de (E.D.); anja.heselich@kgu.de (A.H.); r.sader@em.uni-frankfurt.de (R.S.)

**Keywords:** biomolecular analysis, RNA, histology, methacarn, FFPE, bone tissue

## Abstract

The combination of histological and biomolecular analyses provides deep understanding of different biological processes and is of high interest for basic and applied research. However, the available analytical methods are still limited, especially when considering bone samples. This study compared different fixation media to identify a sufficient analytical method for the combination of histological, immuno-histological and biomolecular analyses of the same fixed, processed and paraffin embedded bone sample. Bone core biopsies of rats’ femurs were fixed in different media (RNAlater + formaldehyde (R + FFPE), methacarn (MFPE) or formaldehyde (FFPE)) for 1 week prior to decalcification by EDTA and further histological processing and paraffin embedding. Snap freezing (unfixed frozen tissue, UFT) and incubation in RNAlater were used as additional controls. After gaining the paraffin sections for histological and immunohistological analysis, the samples were deparaffined and RNA was isolated by a modified TRIZOL protocol. Subsequently, gene expression was evaluated using RT-qPCR. Comparable histo-morphological and immuno-histological results were evident in all paraffin embedded samples of MFPE, FFPE and R + FFPE. The isolated RNA in the group of MFPE showed a high concentration and high purity, which was comparable to the UFT and RNAlater groups. However, in the groups of FFPE and R + FFPE, the RNA quality and quantity were statistically significantly lower when compared to MFPE, UFT and RNAlater. RT-qPCR results showed a comparable outcome in the group of MFPE and UFT, whereas the groups of FFPE and R + FFPE did not result in a correctly amplified gene product. Sample fixation by means of methacarn is of high interest for clinical samples to allow a combination of histological, immunohistological and biomolecular analysis. The implementation of such evaluation method in clinical research may allow a deeper understanding of the processes of bone formation and regeneration.

## 1. Introduction

Bone regeneration is a complex process in which different cell types, signaling molecules and extracellular matrix proteins are involved [1].

The regeneration of bone defects resulting from different diseases, cancer or trauma is often a challenging clinical indication [2,3,4]. Deep research in this field is still needed for many indications such as congenital diseases, osteoporosis, medication related bone necrosis of the jaw or biomaterial-based bone regeneration.

Basic research studies that investigate bone tissue are mainly performed in preclinical models with standardized design and under controlled conditions [5,6,7]. This allows the generation of a sufficient sample size and number to perform different evaluation procedures such as histological analysis, determination of different released signaling molecules and characterization of the gene expression profile, hence enabling a deeper analysis in order to understand the mechanisms of bone formation under different conditions [5].

To translate basic research findings to clinical applications, clinical studies are mandatory. In this case, the amount and size of samples gained from patients are very limited. Additionally, the transport of clinical samples from the operation theater to the analyzing lab is often time consuming and requires high logistic efforts. Therefore, the evaluation of samples gained from clinical studies is often limited to clinical observational outcomes [8], radiological data [9] or single histological tests, if tissue samples are available [10,11]. A deeper evaluation including biomolecular analysis of clinical samples is of high relevance to understand the mechanisms of bone formation or further characterize disease mechanisms [12].

Currently, the gold standard to analyze bone tissue samples from clinical studies is histological analysis [13,14,15]. This technique provides sufficient data about the tissue distribution and the cellular reaction to different treatments. Additionally, immunohistological analysis is becoming more commonly implemented in the evaluation of clinical samples to detect specific proteins expressed by cells residing in the region of interest or to identify the cell type using antibodies to address a specific cluster of differentiation [16]. The application of immuno-histological methods has broadened the field of analysis and allowed understanding of several mechanisms.

Biomolecular analysis of the gene expression is a further method that may elucidate many yet unanswered questions. However, the combination of histological analysis and biomolecular evaluation of clinical samples, especially bone biopsies, is still a very challenging task. One of the most limiting parameters is the number and size of the clinical biopsies that can be gained after bone treatment, for example, prior to the insertion of dental implants [2]. In this context, one possibility of performing histological analysis, as well as gene expression evaluation using clinical samples, is to divide the number of samples gained between the different tests, which reduces the number of analyzed samples and affects the power of statistical analysis. Another limiting factor is the sensitivity of the preservation of RNA in the gained samples. These current limitations highlight the need for more research in optimizing the processing of bone samples with a small size to allow a detailed multidimensional analysis.

Many efforts have been invested in establishing sufficient methods for nucleic acid isolation form bone samples [17]. Introducing methods to isolate RNA from formalin fixed paraffin embedded tissues has opened new paths for diagnostic and therapeutic possibilities in different disciplines [18]. However, formalin fixation was shown to degrade the RNA, which makes a sufficient biomolecular analysis of the same sample very difficult [19]. Recent studies analyzed different fixative media to maintain the histological tissue quality, but avoid the negative effects of formalin on RNA quality. In this context, fixative media such as fresh freezing, or chemical agents such as PAXgen, were shown to allow a more sufficient RNA isolation and gene amplification compared to formalin [20]. Meanwhile, different fixation media are available for sample preparation. For example, RNA-later is used mainly in basic research to fix tissue or cell samples for successful RNA isolation [21]. It was also shown to be a suitable fixative medium for immunohistochemical staining [22]. Another fixative medium, methacarn, was proved to be the most sufficient, especially when performing laser microdissection in decalcified samples [23]. However, more research is needed to establish standardized and effective protocols.

An additional challenge when analyzing bone samples is the need for decalcification prior to histological analysis. This step may also impair the yield RNA quality [24]. Currently, the available evidence on combined histological and biomolecular analysis methods from decalcified bone samples is very limited [25].

Therefore, the aim of the present study was to evaluate different analysis protocols to allow a combined histological, immuno-histological and biomolecular analysis of the same fixed, processed and paraffin embedded bone sample. For this purpose, standardized bone biopsies were treated with different fixation media and evaluated using both histological and biomolecular methods.

## 2. Material and Methods

### 2.1. Sample Collection and Reparation

Eight adult Wistar rats (*n* = 8) were sacrificed with an overdose of ketamine/xylazine from another preclinical study, which was already approved by the responsible regulating authorities of Darmstadt, Germany (FK1023 Regierungspräsidium Darmstadt). The femurs that resulted as waste were used for the present study. All used animals were healthy adult rats that were used as a control group and were hold under the same condition without further manipulation. Per animal, five bone samples 2 mm deep were taken from the distal femoral metaphysis using a 3.2 mm diameter trephine drill, resulting in a sample size of 2 × 3 mm (14 mm^3^).

One of the samples was randomly assigned to the control group of unfixed frozen tissue (UFT) and the other four samples were treated following different fixation and preparation protocols as described in Table 1.

Bone samples were prepared for histological analysis and embedded in paraffin (PE). For demineralization, each sample was incubated in 1 mL of EDTA solution (RNase free) for three days at 4 °C. The samples were then processed manually at room temperature (RT) under RNase free conditions, as follows: 70% ethanol for 45 min, 96% ethanol for 105 min (one change), 100% ethanol for 165 min (two changes), xylene for 135 min (two changes). Finally, the samples were immersed in paraffin wax for 165 min (one change) at 58 °C and embedded in paraffin blocks. The paraffin blocks were stored at 4 °C until RNA extraction.

### 2.2. Histological Analysis

For the histological analysis, five sections of 4-µm thickness were cut from each paraffin block using a rotary microtome (Leica RM2255, Wetzlar, Germany). The sections were attached to positive charged glass slides for better adhesion (SuperFrost^®^ Plus, Menzel Gläser, Thermo scientific, Dreieich, Germany). Prior to staining, the sections were first deparaffinized in xylene and then rehydrated in a descending ethanol series as previously described [26]. Three sections per sample were routinely stained with hematoxylin and eosin (H & E) for basic morphological evaluation. The other two sections were used for immunohistochemistry (IHC). In this study, a murine macro-sialin (CD68) antibody (Mouse Anti-Rat CD68, clone ED1 from Bio-Rad, Hercules, CA, USA) was exemplarily used as previously described [27]. CD68 is considered a cell pan marker for monocytes and macrophages because it is expressed as a surface protein, especially in osteomacs within the bony tissue (Damoiseaux et al. 1994; Miron and Bosshardt 2016). Heat induced epitope retrieval was performed for all samples in a water bath (96 °C for 20 min). Incubation of the sections with CD68 antibody was performed at a ratio of 1:400 for 30 min at room temperature. Detection and visualization of antibody binding was achieved using the UltraVision™ Quanto detection system HRP (Thermo Fisher, Waltham, MA, USA). Counterstaining was performed using Mayer’s Hemalaun. IHC staining was performed manually according to the manufacturer’s protocol. Finally, the samples were embedded with Entellan. A rat bone sample expressing CD-68 from a different independent study was used as a positive control. Additionally, incubation without primary antibody was used as a negative control.

Histo-morphological analysis was performed under the Nikon Eclipse 80i light microscope (Nikon, Japan), and section images were photographed with a microscope digital camera (Nikon DS-Fi1 with Digital Sight Unit DS-L2, Nikon, Japan). The samples were blinded and evaluated by two of the authors (SA and PV) individually. Staining characteristics, tissue architecture, cell morphology and sharpness of the outlines were evaluated on a three-point rating scale ranging from 1 (not preserved), 2 (impaired) to 3 (preserved). The sectional images of the FFPE bone specimens were considered as standard control and thus used as reference.

### 2.3. Biomolecular Analysis

#### 2.3.1. Isolation of Total RNA from Unfixed Frozen Bone Samples

For the unfixed frozen bone samples from the control group UFT, a modified TRIZOL protocol for RNA isolation was performed immediately after sample collection.

All bone samples were first incubated in liquid nitrogen for 10–15 min before being rapidly mechanically crushed with a hammer in an envelope made of sterile foil. The resulting fragments were placed in 1 mL of TRI Reagent^®^ (Sigma, Taufkirchen, Germany) previously precooled on ice, and mixed on a vortex for 10 s. Following the protocol of Cepollaro et. al. 2018 [28] to aid homogenization and cell lysis, samples were first incubated for 15 min at RT and then for an additional 2 h at 4 °C, along with mixing on a vortex after 15 min, 1 h, and 2 h for 10 s each. Isolation and Purification of RNA was performed according to the modified TRIZOL protocol (Supplementary Material Data S1).

#### 2.3.2. Isolation of Total RNA from Fixed and Paraffin Embedded Bone Samples

In case of the one-week fixed bone samples (RNAlater, FFPE, MFPE, R + FFPE), the following pre-treatments were performed prior to RNA isolation. For the PE test groups, the remaining bone samples were removed from the paraffin blocks using a 4 mm diameter tissue punch & excess paraffin was carefully removed. Deparaffinization of the samples was performed as described in supplementary Material Data S2. For the fixed control group RNAlater, the bone samples were simply removed from the storage medium and excess medium was blotted using sterile compresses. Finally, the modified TRIZOL protocol for RNA isolation described in supplementary material Data S1 was also performed on these four fixed samples.

#### 2.3.3. Quantification of RNA and Quality Control

The spectrophotometer NanoDrop 2.000 (Thermo Fisher, Waltham, MA, USA) was used to measure the RNA concentration and contamination level in 1 µL of each RNA solution. The solutions were considered pure if the 260/280 nm and the 260/230 nm ratio were greater than 1.8 [29,30].

To evaluate ribosomal RNA (rRNA) integrity, the isolated RNA was electro-phoretically separated to assess the ratio of the 28S to the 18S band of the rRNA subunits. Immediately after isolation of RNA, gel electrophoresis was performed using 300 ng of total RNA per sample. As a positive control, an appropriate amount of osteoblast RNA previously isolated from a human osteoblast culture was run along with each gel. All RNA solutions were prepared to a total volume of 18 µL with nuclease-free water and 3 µL of loading buffer and incubated at 70 °C for 10 min to break potential secondary structures. The RNA samples were separated onto a 1% agarose gel containing TAE buffer (1X) and SafeView^TM^ Classic RNA/DNA stain (Applied Biological Materials Inc., Richmond, BC, Canada). The gel was analysed using the ChemiDoc XRS + imaging system and Image Lab software (Bio-Rad, Hercules, CA, USA).

#### 2.3.4. Gene Expression

The yield RNA was reverse-transcribed to cDNA using Omniscript RT Kit-Master Mix (Qiagen, Hilden, Germany) and randomized hexamer primers (Qiagen, Hilden, Germany) according to the manufacturer’s instructions. For quantitative real-time polymerase chain reaction (qPCR), primers were designed as single-stranded 25 nmole DNA oligonucleotides based on the U.S. National Center for Biotechnology Information (NCBI) nucleotide database and obtained from Integrated DNA Technologies (Skokie, IL, USA) (Table 2). Thereby collagen type-I α-1 (Col1a1) was selected as target gene, whereas B2M (β-2 micro-globulin) and PPIB (peptidyl-prolyl isomerase B) were selected as reference genes based on geNorm and Normfinder analysis. qPCR amplification was performed with the StepOnePlus real-time PCR system (Applied Biosystems, Foster City, CA, USA). Each reaction mix with a total volume of 20 µL contained 10 µL of 2x SYBR^®^ Green JumpStart™ Taq ReadyMix™ (Sigma-Aldrich, St. Louis, MO, USA), 0.9 µL each of forward and reverse primers (10 µM), 4.2 µL of aqua ad injectabilia and 4 µL of the respective cDNA solution (diluted to 1 ng/µL) or aqua ad injectabilia (NTC). The experiments were performed in triplicates. Initial heat activation (94 °C, 2 min) was followed by 40 cycles of denaturation (94 °C, 15 s), annealing and elongation (60 °C, 1 min).

The ΔCt method was calculated in relation to the reference genes for each sample:

$$\Delta \mathrm{Ct}\;{\left(\mathrm{target}\;\mathrm{gene}\right)}_{i}=\mathrm{Ct}\;{\left(\mathrm{target}\;\mathrm{gene}\right)}_{i}-\;\mathrm{Ct}\;{\left(\bar{\mathrm{reference}\;\mathrm{genes}}\right)}_{i}$$

i: respective group, $\mathrm{Ct}\;{\left(\bar{\mathrm{reference}\;\mathrm{genes}}\right)}_{i}$: mean of reference genes.

For visualization, after the qPCR run 10 µL of each qPCR reaction solution was diluted with 5 µL aqua ad injectabilia and 3 µL DNA Dye Nontox (PanReac AppliChem, Darmstadt, Germany) and applied to a 2.5% agarose gel containing TAE buffer (1X).

### 2.4. Statistical Evaluation

Statistical analysis was performed on the data obtained from NanoDrop analysis and Ct values from the qPCR of all five groups (with *n* = 8 samples per group). The arithmetic mean (ø) and standard deviation (±SD) were used to evaluate the statistical significance by a simple analysis of variance (ANOVA) followed by Tukey’s test, performed using the program GraphPad Prism version 8.0.1 (GraphPad Software, La Jolla, CA, USA). The statistical significance level was set at *p* < 0.05 and marked with (*). Differences with a *p* value < 0.01 were considered very significant (**) and with *p* < 0.001 highly significant (***). The graphical representation of the results was in the form of a bar or box-whisker plot, also by the GraphPad Prism program. The cross bar in the middle of the box indicates the median.

## 3. Experiment Results

### 3.1. Histological and Immunohistological Evaluation

**Figure 1:**
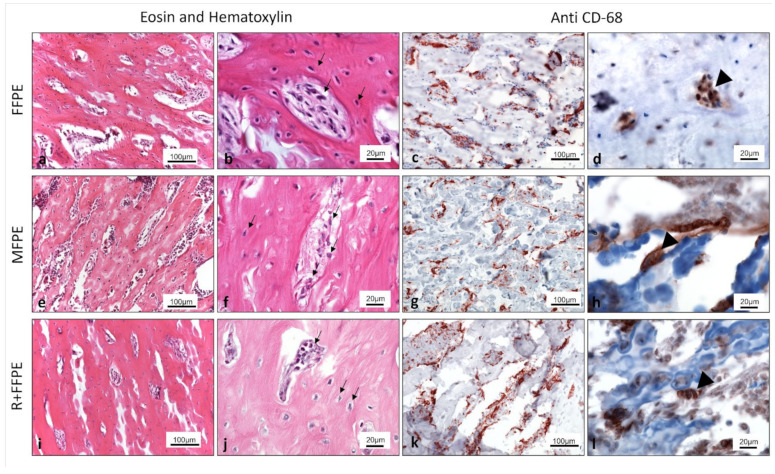
Histological and immunohistological analysis of the differently treated bone samples showing comparable histo-morphological results as demonstrated by H & E staining and positive immunohistological staining as demonstrated by the anti-CD 68 staining. Comparable cell morphology and structure sharpness was observed in the groups of FFPE and MFPE. However in the group of R + FFPE some loss of fixation quality was observed, reflected by the irregular and less distinguishable cell margins. CD-68 staining showed positively stained cells in all groups labeled as the so called osteomacs, a special type of macrophages found within the bony tissue. (**a**–**d**) formaldehyde fixed (FFPE) paraffine embedded samples, (**e**–**h**) methacarn fixed paraffin embedded samples (MFPE), (**i**–**l**) RNAlater and formaldehyde fixed paraffine embedded samples (R + FFPE). Arrows point to osteocytes within the osteocytes lacunae and mononuclear cells within the intertrabecular area. Arrow heads point to CD-68 positive cells.

Qualitative evaluation by light microscopy focused on the morphology of the stained structures and cells. The FFPE group was used as a standard control for histology and showed intact bone structure including clearly observable cells displaying the eosinophilic cytoplasm and the basophilic nuclei as documented by the H & E staining. Additionally, in this group the structures were sharp and highly distinguishable. In comparison, the groups of MFPE and R + FFPE showed in the H & E staining a well-preserved bone structure and adequately stained cells (eosinophilic and basophilic parts). However, in the group of R + FFPE a slight loss of quality was observed, especially when evaluating the margins of single structures, that appeared rather blurred and were less well preserved compared to the other groups. The immunohistochemical staining showed a positive and structure-specific staining in all groups. CD 68 positive cells were exhibited all over the samples. These cells are considered a special type of macrophage residing in the bone tissue (Table 3), (Figure 1a–l).

### 3.2. Biomolecular Evaluation

Biomolecular evaluation included the RNA quantity and quality as well as the gene expression as described below.

#### 3.2.1. Extracted RNA from Differently Treated Samples

**Figure 2:**
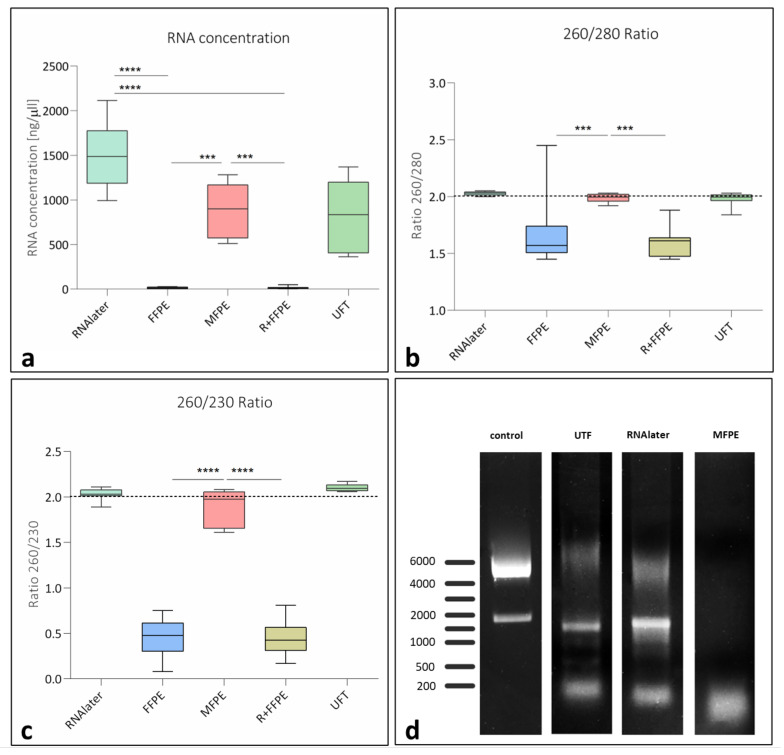
Qualitative and quantitative RNA isolation results. (**a**) The yield RNA in differently treated samples, (**b**) the 260/280 ratio of differently treated samples, (**c**) the 260/230 ration of differently treated samples, (**d**) gel electrophoresis of the yield RNA from differently treated samples. FFPE: formaldehyde fixed paraffin embedded samples, MFPE: methacarn fixed paraffin embedded samples, R + FFPE: RNAlater and formaldehyde fixed paraffine embedded samples. Statistical differences are presented as *** *p*< 0.001 and **** *p* < 0.0001.

The RNA quantity yield was the highest in the group of RNAlater. A comparable amount of RNA was extracted from the UFT group without statistically significant difference compared to RNAlater. Interestingly, MFPE showed the highest extracted RNA quantity among the fixed and paraffin embedded test groups showing comparable value to the groups of RNAlater and UFT without statistically significant differences. By contrast the other two fixed and paraffin embedded groups FFPE and R + FFPE resulted in a very low RNA yield that was statistically significantly lower compared to MFPE and RNAlater (*** *p* < 0.001 for both), (Figure 2a).

Considering the RNA quality, the 260/280 ratio was higher than 1.8 in the groups of RNAlater, UFT and MFPE without statistically significant differences. However, from the processed and paraffin embedded groups only MFPE showed a 260/280 ratio above 1.8 that was statistically significantly higher compared to the groups FFPE and R + FFPE (** *p* < 0.01 for FFPE and *** *p* < 0.001 for R + FFPE), (Figure 2b). Moreover, the 260/230 ratio showed a comparable pattern to that of the 260/280 ratio. Here, the group of MFPE was the only fixed test group that showed a comparable ratio to the unfixed groups of RNAlater and UFT. It was also statistically significantly higher compared to FFPE and R + FFPE (**** *p* < 0.0001 for both), (Figure 2c).

However, the evaluation of the ribosomal RNA (rRNA) integrity using gel electrophoresis showed two clearly distinguishable bands with a 1:2 ratio for the unfixed groups UFT and RNAlater that were comparable to the positive control (osteoblasts), but not in the MFPE group. No gel electrophoresis could be performed for the other two fixed groups FFPE and R + FFPE, due to the lack of RNA quantity extracted (Figure 2d).

#### 3.2.2. Gene Expression

**Figure 3:**
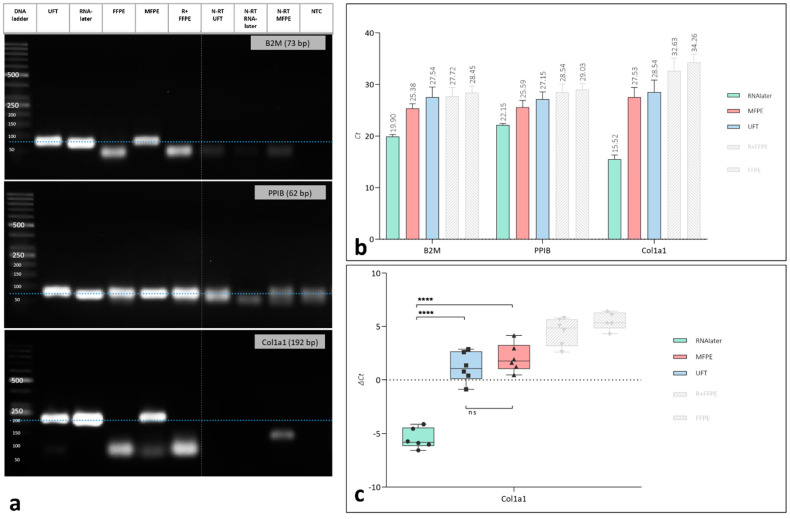
Gene expression analysis by RT-qPCR of the differently treated samples. (**a**) Visualization of the amplified genes in the differently treated groups by means of gene electrophoresis. (**b**) The Ct-values of the used genes B2M, PPIB and Collagen 1. (**c**) The ΔCt-values of the differently treated samples. FFPE: formaldehyde fixed paraffin embedded samples, MFPE: methacarn fixed paraffin embedded samples, R + FFPE: RNAlater and formaldehyde fixed paraffin embedded samples, bp: base pairs. Statistical differences are presented as **** *p* < 0.0001, n.s.: not significant.

Gel electrophoresis was performed after qPCR to assess whether the respective gene product was amplified correctly. In this case, the correct gene product was adequately localized on the agarose gel in the groups of RNAlater, MFPE and UFT, especially for the genes B2M and Col1a1, whereas the gene products of these two evaluated genes were not identifiable in the groups of FFPE and R + FFPE. In the case of PPIB, the bands on the gel were sharply identifiable in the groups of RNAlater, MFPE and UFT. However, the bands in the groups of FFPE and R + FFPE were adequately localized but not sharply distinguishable (Figure 3a).

The evaluation of the gene expression was performed using the Ct-values and the ΔCt-values. The Ct-values for each analysed gene (reference and target genes) was compared between the evaluated examples. Interestingly, for all three evaluated genes the Ct-values were the lowest in the group of RNAlater, followed by the group of MFPE and UFT. By contrast, the groups R + FFPE and FFPE showed the highest Ct-values (Figure 3b).

The ΔCt-value was the highest in the groups of FFPE and R + FFPE. However, in the group of MFPE the measured ΔCt-value was comparable to that of the UFT group without any statistically significant difference. Interestingly, the ΔCt-value of the RNAlater group was the lowest and showed statistically significantly lower value when compared to MFPE and UFT (*** *p* < 0.001 for both), (Figure 3c).

## 4. Discussion

The use of different analysis methods to evaluate bone formation and regeneration allows a deeper understanding of pathophysiological mechanisms and evaluation of treatment techniques [31,32]. Thus, the combination of classical methods of histology with biomolecular analysis techniques is of high interest for both basic and applied research fields. However, some limitations are currently still being faced, especially when considering clinical bone biopsies, including the limited size and number, as well as transportation and storage methods. These limitations highlight the need for developing clinically applicable protocols to facilitate multidisciplinary analysis. Therefore, the present study aimed to evaluate different fixation and preparation protocols to introduce an optimized analysis method enabling the histochemical, immuno-histological and biomolecular analysis of the same fixed, processed and paraffin embedded bone sample.

In the present study, cylinder core bone biopsies with 3 mm diameter and 2 mm length were obtained from the femur of Wistar rats as a model for core biopsies to represent the clinical procedure applied in the field of oral and maxillofacial surgery [2]. The study aimed to simulate the complete procedure for clinical samples including fixation, transport, histological processing and storage. In this context, the samples were incubated in different fixation media (RNAlater (R + FFPE), methacarn (MFPE) and formaldehyde (FFPE)) for 1 week prior to further processing to consider the transportation time from the clinic to the evaluation lab. Thereafter, the samples were decalcified, processed and embedded in paraffin following a standard procedure for histo-morphological evaluation of bone samples. Two control groups were considered in this study. First, snap-freezing using liquid nitrogen (UFT), which is a very reliable method to preserve the RNA for biomolecular analysis. It is also possible to use this method for further histological analysis using cryo-sectioning. However, this method is not easily accessible for clinical samples, as liquid nitrogen is usually not a routine medium for clinical application, and cryo-sectioning is not always available. Additionally, transport and storage displays another limitation for this method, as they have to be performed at −80 °C until evaluation (Son, Sokolowski, and Zhou 2013). A second control group was included using RNAlater, as a reliable medium for RNA fixation, without further processing and embedding, to be used as a reference for the R + FFPE group [22].

The histological analysis of the fixed and processed samples documented by H & E staining showed adequate results concerning the staining quality and structure preservation in both groups, MFPE and R + FFPE, compared to the gold standard FFPE, although a slight loss of quality in the sharpness of the structures was observed in the group of R + FFPE (Table 3). Additionally, immunohistological staining was successfully performed in all groups. Here, CD-68 was used as a marker for osteomacs [33], which are a special type of macrophage found in bony tissue. The results showed that both methacarn and RNAlater can be used as fixation media for histological and immunohistological analysis and deliver comparable results to formaldehyde. Our results are in agreement with previously published studies analyzing soft tissue. For example two studies demonstrated sufficient results when using methacarn for fixing biopsies of rats liver [34] or muscle tissue [35] with comparable results to formaldehyde. In comparison to these studies, the bone samples used in our study provide more challenges, because they normally consist of a dense mineralized extracellular matrix with less cellular density compared to the soft tissues. Therefore, in our case decalcification with EDTA was performed in addition to the standard histological processing to allow tissue sectioning. Only very few studies were found in the literature that analyzed different preparation protocols for bone tissue including decalcification [25]. In a previous study, cochlea samples were fixed using methacarn combined with different decalcification media. The authors concluded that the combination of methacarn as fixation medium with EDTA-decalcification is a sufficient protocol for histological analysis [25]. Besides, it is important to note, that previously published studies of the literature mostly used methacarn fixation for a maximum of 3 days. In our study, the incubation in methacarn lasted for 7 days. Thereby the present study additionally showed that a longer fixation does not impair the histolo-morphological preservation quality. Interestingly, methacarn was reported to allow improved immunohistological staining quality when compared to formaldehyde [35]. This finding was not precisely observed in our study.

The results of the biomolecular analysis achieved a relatively high concentration of RNA gained from the MFPE group that was comparable to the unfixed groups of UFT and RNAlater. However, it was significantly higher compared to the other two fixed and paraffin embedded groups of FFPE and R + FFPE. These outcomes are similar to previously reported results in the literature evaluating unmineralized tissue fixed using methacarn and showing comparable results to those of snap freezing [36,37]. However, in our study, the tissue was not only fixed and paraffin embedded, but also decalcified by EDTA. In this context, the EDTA decalcification performed using the protocol suggested here did not affect the RNA preservation negatively. Another study analyzed the effect of fixation media including methacarn using rat mandible samples that were additionally decalcified using EDTA (Salmon et al. 2012). The authors compared the RNA yield to unfixed frozen liver samples as a control. They reported that methacarn fixed and EDTA decalcified mandible samples resulted in higher amount of RNA when compared to formaldehyde fixation, but less when compared to fixed frozen liver samples [38]. In this case a direct comparison of the same tissue type (i.e., mandible) was not performed [38]. By contrast, in our study bone samples from the same rat bone, either fixed using methacarn or by snap freezing, showed a similar amount of RNA without statistically significant differences. However, when comparing the used protocols, some differences between the methods used by Salmon et al. [38] and our protocol became clear. One effect might have occurred during the fixation. Salmon et al. fixed their samples in methacarn for 2 h, whereas our protocol considered a fixation for 1 week. Therefore, the short time of 2 h might not have been sufficient to achieve full fixation of the dense mineralized bone samples. Additionally, Salmon et al. 2012 decalcified their samples for 15 days, whereas our decalcification lasted for 3 days, which might have also led to the observed differences in the amount of extracted RNA. These hypotheses are supported by a study that suggested switching the steps of fixation and decalcification, suggesting first decalcification of bone samples before fixing them [39]. The results showed a better preservation of the RNA when starting with the decalcification [23]. Therefore decalcification leads to a removal of the dense mineralization and allows a faster and easier penetration of the fixative medium into the samples, resulting in a better preservation of the RNA [39]. Alternatively, a longer fixation period to sufficiently penetrate the samples seems to be mandatory to achieve adequate RNA preservation in bone samples.

In addition to the RNA quantity, the quality of the RNA yield plays an important role for the subsequent biomolecular analysis. Our results showed a very good RNA quality in the MFPE group (1.99 and 1.89) that was comparable to UFT and RNAlater, whereas the FFPE and R + FFPE showed a significantly lower quality when considering the 260/280 and 260/230 ratio. Similar results showing such high purity were not found in the literature at this time point. However, previous studies reported a sufficient RNA quality with ratios ranging from 1.0 to 1.32, when soft tissue samples were fixed using [35,36]. The different results in comparison to our study may be explained by the different fixation periods, that lasted for 2 days in the described studies and one week in our study. Moreover, the used RNA isolation protocols used in the described studies also differed from our modified TRIZOL-based protocol (Supplementary material Data S1).

The integrity of the isolated RNA was the highest in the groups of UFT and RNAlater that did not undergo decalcification or paraffin embedding. In comparison, the group of MFPE did not show a defined 2:1 ratio of the 28S/18S bands as documented by gel electrophoresis. No comparison with the groups of FFPE and R + FFPE is possible because these groups did not provide enough RNA to perform gel electrophoresis. These results showed that, despite the high amount of the isolated RNA from the MFPE group and the high purity, the RNA integrity was affected by the fixation, decalcification, processing and paraffin embedding. Similar results were also reported in previous studies showing the lack of RNA integrity in the methacarn fixed and paraffin embedded samples [23,38]. RNA integrity is an important parameter when performing bimolecular analysis. However, the here evaluated 28S/18S ratio is a criteria that applies for the ribosomal RNA (rRNA) and does not necessarily provide sufficient information about the integrity of the mRNA [39,40]. Therefore, gene amplification using the isolated mRNA may still be useful depending on the target genes for each scientific question.

In this context, in our study the isolated total RNA of all groups was used for RT-qPCR regardless of the results of the gel electrophoresis (rRNA integrity). Gel electrophoresis of the amplified genes showed clearly and identifiable gene product that was only correctly amplified in the groups of RNAlater, UFT and MFPE. Similar results were documented by previous studies showing an affected RNA integrity but positive RT-qPCR results, especially when using methacarn as a fixative medium [23,38]. In contrast, the groups of FFPE and R + FFPE did not show a correctly amplified gene product at the respective expected base pair length. The results of the RT-qPCR in this case may be related to unspecific amplification for example by mis-priming that occurs especially with increasing cycle numbers [41] or genomic DNA contamination.

Based on the described findings, the base pair length of the addressed target gene became of high importance when using compromised RNA. Thereby, the integrity of the isolated RNA has to be sufficient to amplify the targeted qPCR product. In our case, a rather small amplicon (Col1a1) with a base pair length of 192 bp was targeted. Other studies showed successful amplification of similar gene products such as BMP-2 (69 bp) or Runx (395 bp) ([23,38] after decalcification followed by methacarn fixation and paraffin embedding. Additionally, it was shown that a successful amplification of the qPCR products with a base pair length up to 600 bp from methacarn fixed samples is possible despite of the affected integrity [35,36,37,42,43].

Interestingly, the RT-qPCR results showed similar Ct-values in the groups of UFT, RNAlater and MFPE. In contrast, the groups of FFPE and R + FFPE showed higher Ct-Values, which are not comparable with the other groups, because the target gene was not amplified correctly. These results are consistent with the quantity and quality of the isolated RNA as documented by the spectrophotometric analysis. Consequently, the calculated ΔCt-values were comparable in the groups of MFPE and UFT when analyzing the expression of Col1a1. In the present study the calculated ΔCt-values were used instead of the calculated ΔΔCt-values, because no treatment was performed that was expected to influence the RNA expression. Therefore, no treatment control is available to calculate ΔΔCt-values. Based on the addressed question and the used native bone samples ΔCt-values were found to be sufficient to evaluate the gene expression at this stage. Further studies using native and treated bone may be necessary to validate the presented results.

The demonstrated results may be explained by the known mechanisms of tissue fixation using formaldehyde in comparison to other fixatives. Formaldehyde leads to crosslinking of the nucleic acids during two reactions including an addition of N-methylol and an electrophilic attack to form a methylene bridge between two amino groups. Specifically the adenine base is affected by this reaction leading to a heavily modified poly-A tail of the treated mRNA [20,44]. Consequently, the extracted RNA is degraded and cDNA synthesis as well as gene amplification are highly compromised. By contrast, methacarn is a non-cross-linking fixative consisting of methanol, 60%; chloroform, 30%; and acetic acid, 10%. The nucleic acids such as DNA and RNA were shown to collapse in alcoholic environment without affecting the initial confirmation or the primary structure [43,45]. Other explanations were attributed to the precipitation of ribosomal proteins by methacarn and the inactivation of the endogenous RNAse, which prevents RNA degradation. Another reason may be inactivating the residual RNase by methacarn resulting in protecting the mRNA from degradation [44,46].

Altogether, the results of the present study introduced a possibility of performing combined histological, immunohistological and biomolecular analysis using the same decalcified and paraffin embedded bone sample. The RNA isolated by using methacarn fixation followed by EDTA decalcification was shown to be sufficient for analysis and to meet the most important clinical requirements. The suggested protocol allows its implementation in the clinical routine to enable multidisciplinary analysis from valuable clinical samples of limited size. One possibility for the implementation of such a method may be the analysis of samples resulting from treatment with bone substitute materials or tissue engineering scaffolds. This may enhance the multidisciplinary analyses of clinical samples and gain a deeper understanding of bone mechanisms, for example, in the field of biomaterials-based bone regeneration and tissue engineering.

## 5. Conclusions

The present study evaluated different fixation media to identify an effective analysis protocol that enables the histological, immunohistological and biomolecular analysis of the same fixed, decalcified, processed and paraffin embedded bone sample. The results showed that a one-week fixation using methacarn, followed by EDTA decalcification for 3 days, allows comparable histological and immunohistological results to the formaldehyde fixed samples, and enables RNA isolation with a high quantity and quality that is comparable to snap frozen samples. This method is promising for implementation in clinical studies with limited sample size.

## Figures and Tables

**Table 1:** Overview of the group categories. P + PE = processed and paraffin-embedded, UFT = unfixed frozen tissue, FFPE = formaldehyde-fixed paraffin-embedded, MFPE = methacarn-fixed paraffin-embedded (freshly prepared methacarn consists of 60% methanol, 30% chloroform, 10% acetic acid), R + FFPE = RNAlater-incubated + FFPE.

Group	Category	Fixation Medium	Incubation Time	P + PE
**UFT**	control	snap-frozen (in liquid nitrogen)	15 min	-
**RNAlater**	control	RNAlater	1 week	-
**FFPE**	test	Formaldehyde (Roti^®^-Histofix)	1 week	+
**MFPE**	test	Methacarn	1 week	+
**R + FFPE**	test	RNAlater (6 days) + formaldehyde (Roti^®^-Histofix) (24 h)	1 week	+

**Table 2:** Primer design and specifications.

Gene	NCBI Accession Number (mRNA)	mRNA Length (bp)	5′-Forward-Primer-3′ 5′-Reverse-Primer-3′	Primer Length (bp)
**Reference Genes**				
**B2M**	NM_012512.2	1845	TCTCTCTGGCCGTCGTGCTT	20
			TTCTCCGGTGGATGGCGAGA	20
**PPIB**	NM_022536.2	838	ACG TGG TTT TCG GCA AAG T	19
			CTT GGT GTT CTC CAC CTT CC	20
**Target Gene**				
**Col1a1**	NM_053304.1	5843	CCTGACGCATGGCCAAGAAG	20
			CACTCGCCCTCCCGTTTTTG	20

**Table 3:** Criteria for histological evaluation using a three-point rating scale ranging from 1 (not preserved), 2 (impaired) to 3 (preserved).

Criteria	FFPE	MFPE	R + FFPE
**Staining characteristics**	3	3	3
**Tissue architecture**	3	3	3
**Cell morphology and sharpness**	3	3	2
**Total**	9	9	8

## Data Availability

The data presented in this study are available on request from the corresponding author.

## Supplementary Materials

The following supporting information can be downloaded at: https://www.mdpi.com/article/10.3390/mps5040064/s1, A detailed description of the used protocols are found in the supplementary Data S1: modified TRIZOL Protocol for RNA isolation and purification; Data S2: Protocol for sample deparaffinization prior to RNA isolation and purification.
