# Supplementary material for: Comparison of Different Fixation Methods for Combined Histological and Biomolecular Analysis of Fixed and Decalcified Bone Samples
Source: Methods Protoc. 2022 Jul 21;5(4):64. doi: 10.3390/mps5040064 (PMC9326524; doi:10.3390/mps5040064)
Supplement: Supplementary file 1 [file mps-05-00064-s001.zip › SupplementalData-S1.pdf]

**Supplementary material S1: modified TRIZOL Protocol for RNA isolation and purification**

to aid homogenization and cell lysis, samples were first incubated for 15 min at RT and then for an additional 2 h at 4 °C, with mixing on a vortex after 15 min, 1 h, and 2 h for 10 sec each. Purification of RNA was performed according to the following protocol:

(1) Adding 200 µl of chloroform and vortex for 10 sec.

(2) 15 min incubation on ice.

(3) Centrifugation at 12,000 rpm for 15 min at 4 °C.

(4) Transferring the aqueous phase containing RNA (about 450 µl) to a new reagent tube.

(5) Adding 500 µl of isopropanol and mixing carefully by inverting.

(6) 10 min incubation on ice.

(7) Centrifugation at 12,000 rpm for 10 min at 4 °C. The isopropanol caused precipitation of the RNA molecules and a nucleic acid pellet was deposited on the wall of the reagent tube.

(8) Pipetting off the excess and disposing it. Next, a treatment with the enzyme neutral deoxyribonuclease (DNase I) was performed. Here, Qiagen's RNase-Free DNase Set (Qiagen, Hilden, Germany) was used and the manufacturer's protocol for DNase treatment of RNA in solution was followed, increasing the incubation time from 10 to 15 min to obtain a higher level of digestion.

(9) Dissolution of the pellet in 87.5 µl of nuclease-free water to achieve a reaction volume of 100 µl.

(10) Adding 10 µl of RDD Buffer and 2.5 µl of DNase stock I solution. The solutions were mixed by careful finger flicking and brief centrifugation.

(11) 15 min incubation at RT. Subsequently, to inhibit the DNase I enzymes, a new phase separation was performed with acid phenol and chloroform in a 1:1 ratio.

(12) Adding 50 µl of TRI Reagent and 50 µl of chloroform. Mixing by careful shaking.

(13) Centrifugation at 7,200 rpm for 10 min at 4°C.

(14) Transferring the top transparent phase (about 100 µl) to a new reagent tube.

(15) Adding 10 µl of 3M sodium acetate (pH 5.2) and 250 µl of 100% ethanol. Mixing by finger flicking.
(16) 1.5 h incubation at -20°C.

(17) Centrifugation at 13,300 rpm (max. speed) for 10 min at 4 °C to concentrate the RNA molecules into a pellet. Finally, a purification step was performed with ethanol.

(18) Adding 1 ml of 75% ethanol and detachment of the RNA pellet by finger flicking.

(19) Centrifugation at 13,300 rpm (max. speed) for 5 min at 4 °C.

(20) Pipetting off the excess and disposing it.

(21) Air drying under fume hood for 20 min.

(22) Elution of the pellet in 10 µl of RNase-free water.

(23) Detachment of the RNA pellet by careful finger flicking.

(24) Brief centrifugation (accelerated once to 13,300 rpm) to collect the liquids adhering to the walls. The RNA solutions were stored on ice until the subsequent spectrophotometric measurement.
