# Supplementary material for: Comparison of Different Fixation Methods for Combined Histological and Biomolecular Analysis of Fixed and Decalcified Bone Samples
Source: Methods Protoc. 2022 Jul 21;5(4):64. doi: 10.3390/mps5040064 (PMC9326524; doi:10.3390/mps5040064)
Supplement: Supplementary file 1 [file mps-05-00064-s001.zip › SupplementalData-S2.pdf]

Supplementary material S2: Protocol for sample deparaffinization prior to RNA isolation and purification

(1) Adding 1 ml of xylene.

(2) Vortex for 10 sec.

(3) Incubation for 5 min at 50 °C.

(4) Centrifugation at 13,300 rpm (max. speed) for 2 min at RT.

(5) Incubation for 5 min on ice.

(6) Pipetting off the excess, which contained a thin paraffin layer on the surface, from top to bottom.

(7) Repeating steps (1)-(6).

(8) Adding 1 ml of 100% ethanol.

(9) Vortex for 10 sec.

(10) Centrifugation at 13,300 rpm (max. speed) for 2 min at RT.

(11) Pipetting off the excess.

(12) Repeating steps (8)-(11).

(13) Air drying under the fume hood for 20 min.
